# 1595. Accumulating Antiretroviral Therapy Safety Data In Pregnancy: Barriers To Clinician Reporting To The Antiretroviral Pregnancy Registry In North America And Internationally

**DOI:** 10.1093/ofid/ofad500.1430

**Published:** 2023-11-27

**Authors:** M Stavroula manta, Jessica O’Bryan, Michelle Giles, William R Short, Erin logue-Chamberlain, sushena Krishnaswamy

**Affiliations:** Monash Health, Melbourne, Victoria, Australia; Monash Health, Melbourne, Victoria, Australia; Monash University, Melbourne, Victoria, Australia; University of Pennsylvania, PA; University of Pennsylvania, PA; Monash Health, Melbourne, Victoria, Australia

## Abstract

**Background:**

Clinical trials evaluating antiretroviral therapy (ARV) often exclude pregnant women, delaying the accumulation of safety data in pregnancy, and use of newer ARVs in women of reproductive age. The Antiretroviral Pregnancy Registry (APR) is the largest international exposure registry of ARV use in pregnancy, but only 0.5% of reports come from Australia and 70% of reports are from the United States (US). A decline in reporting was noted during the COVID-19 pandemic.

**Methods:**

Surveys were developed independently and disseminated to clinicians who provide care to women with HIV or their children through email distribution lists of the peak infectious diseases societies in Australia and the US Reproductive Infectious Diseases and HIV email distribution list. The surveys assessed awareness of the APR and barriers to contributing data.

**Results:**

In total, 146 clinicians (80 Australia, 66 US) completed the surveys. Of US respondents, 59% (39/66) used the APR clinically and 55% (36/65) had contributed data to the APR. The key barriers to reporting included lack of time, funding and staff/administrative support, and complexity of the reporting system. In contrast, in Australia, less than half (47.5%, 38/80) of respondents were aware of the APR, and only 10% (8/80) had ever contributed data. The perceived barriers to contributing data in this context were a lack of knowledge (unaware that clinicians outside the US could report, uncertainty about how to report, belief that only adverse pregnancy outcomes should be reported) and feeling their individual contribution would be insignificant when providing care for small numbers of pregnant women. Clinicians in both countries identified consent and ethics review board requirements as barriers to contributing data.

**Conclusion:**

The barriers to reporting data to the APR are likely context specific. The findings from Australia may apply to other high resource settings. Further efforts are required to address these barriers to increase reporting of ARV use in pregnancy. This would increase the acquisition of ARV pregnancy safety data and address the disparity in treatment options faced by women of reproductive age with HIV.

**Disclosures:**

**Stavroula mantaM, B Pharm**, Gilead: Received funding for project: BRAVVO **Michelle Giles, MBBS PhD**, Gilead: Grant/Research Support **William R. Short, MD**, Gilead Sciences: Advisor/Consultant|ViiV: Advisor/Consultant|ViiV: Honoraria **sushena Krishnaswamy, MBBS**, Gilead: Grant/Research Support|Pfizer: Advisor/Consultant

